# Utility of the Fragility Score (FS) Determined Through Radiofrequency Ecographic Multi-Spectrometry (REMS) in the Follow-Up of Patients with Axial Spondyloarthritis (AxSpA)

**DOI:** 10.3390/jcm14072372

**Published:** 2025-03-30

**Authors:** Ionuț-Andrei Badea, Mihai Bojincă, Violeta Bojincă, Mihaela Milicescu, Gabriel Ghițescu, Negoiță Casandra, Andreea-Ruxandra Ilina, Mădălina-Ștefania Vulcan, Ștefan-Sorin Aramă

**Affiliations:** 1 Department of Rheumatology, Carol Davila University of Medicine and Pharmacy, 050474 Bucharest, Romania; mihai.bojinca@umfcd.ro (M.B.); violeta.bojinca@umfcd.ro (V.B.); mihaela.milicescu@umfcd.ro (M.M.); 2 Department of Internal Medicine, Clinical Hospital Dr. I. Cantacuzino, 030167 Bucharest, Romania; 3 Department of Internal Medicine, Sf. Maria Clinical Hospital, 011172 Bucharest, Romania; 4 Osteodensys Private Clinic, 023677 Bucharest, Romania; gabi.ghitescu@genodynamic.ro (G.G.); office@osteodensys.ro (N.C.); 5 Department of Internal Medicine, Colentina Clinical Hospital, 020125 Bucharest, Romania; ruxandrailina@gmail.com (A.-R.I.); madalina-stefania.vulcan@rez.umfcd.ro (M.-Ș.V.); 6 Department of Physiopathology, Carol Davila University of Medicine and Pharmacy, 050474 Bucharest, Romania; sorin.arama@umfcd.ro; 7 Department of Physiopathology, Prof. Dr. Matei Bals National Institute of Infectious Diseases, 021105 Bucharest, Romania

**Keywords:** Radiofrequency Echographic Multi-Spectrometry, fragility score, axial spondylarthritis, vitamin D supplementation, muscle strength

## Abstract

**Abstract: Objectives**: Bone mineral density (BMD) variation under vitamin D supplementation, determined using dual-energy X-ray absorptiometry (DXA), is the gold standard and the main tool used in most studies in this domain. However, the scientific literature is lacking with regard to the usefulness of REMS in BMD follow-up, especially the importance of the fragility score (FS). The main objective of this study was to determine whether FS follow-up is relevant in a group of patients with axial spondyloarthritis and whether REMS could have clinical applicability. **Methods**: Patients with a certain diagnosis of axial spondyloarthritis (AxSpA) were recruited from two medical healthcare centers and were scanned using Radiofrequency Echographic Multi-Spectrometry in order to obtain their fragility score (FS), an objective measurement of bone quality. The main group was randomized into a vitamin D supplementation branch and a non-supplementation branch and followed up every 6 months for 18 months in total. Comparisons between the branches were made using MiniTab v.20 statistical software. **Results**: Lower FS values were obtained in patients who initially had high scores, suggesting a positive impact of vitamin D on bone quality (*p* = 0.008). Muscle strength was evaluated through a visual analogue scale (VAS), with improvements being seen in the supplementation branch (*p* < 0.005). Furthermore, although some patients had experienced falls in previous years, during the study period, no new events were recorded in either group. **Conclusions**: The FS is a reliable tool for evaluating bone architecture and is useful in everyday practice for the management of patients taking vitamin D supplements.

## 1. Introduction

Osteopenia and osteoporosis are skeletal disorders defined by a reduction in bone mineral density (BMD) and deterioration of bone microarchitecture, leading to an increased risk of fractures [1]. Axial spondyloarthritis (axSpA) is a chronic inflammatory condition primarily affecting the axial skeleton. The systemic inflammatory response observed in axSpA disrupts the delicate balance between bone resorption and formation, resulting in the paradoxical coexistence of new bone formation (e.g., syndesmophytes and enthesophytes) alongside generalized bone loss—particularly in trabecular bone of the proximal femur and spine [2,3]. Given vitamin D’s central role in calcium metabolism and immune modulation, its potential therapeutic application in these conditions has attracted significant interest. Understanding the molecular and cellular mechanisms underlying vitamin D’s actions is key to clarifying how supplementation might counteract the processes that lead to bone fragility and abnormal remodeling.

Vitamin D is primarily synthesized in the skin under ultraviolet B (UVB) exposure and is also obtained from dietary sources [4]. In the liver, cholecalciferol is hydroxylated to form 25-hydroxyvitamin D [25(OH)D]—the major circulating form—and further hydroxylated in the kidneys to produce 1,25-dihydroxyvitamin D (calcitriol), the physiologically active hormone [5]. Calcitriol binds to the intracellular vitamin D receptor (VDR); after heterodimerizing with the retinoid X receptor (RXR), it translocates to the nucleus and binds to vitamin D response elements (VDREs) in target gene promoters. This regulates the transcription of proteins that are crucial for intestinal calcium absorption (e.g., TRPV6, calbindin-D₉K), bone matrix formation, and parathyroid hormone (PTH) regulation [6]. Moreover, calcitriol modulates the RANK/RANKL/osteoprotegerin (OPG) system (by upregulating OPG expression, it helps restrain osteoclastogenesis and subsequent bone resorption) while simultaneously enhancing osteoblast differentiation and function to promote bone matrix deposition and mineralization [7].

When vitamin D levels fall below optimal thresholds, several deleterious processes occur:

- Secondary hyperparathyroidism: Inadequate vitamin D reduces intestinal calcium absorption, leading to hypocalcemia. The resulting compensatory increase in PTH (secondary hyperparathyroidism) stimulates osteoclast activity to mobilize calcium from bone, chronically driving excessive bone resorption and increasing fracture risk [8].
- Impaired bone matrix formation: Deficient VDR activation reduces the transcription of genes responsible for synthesizing key bone matrix proteins, resulting in a suboptimal collagen framework and poor-quality bone that is more susceptible to fractures [9].
- Altered osteoblast–osteoclast coupling: Insufficient calcitriol impairs osteoblast function, while unopposed PTH action enhances osteoclast-mediated resorption, contributing to the progression from osteopenia to osteoporosis [10].

Axial spondyloarthritis is marked by a dual pathology: inflammatory cytokines (e.g., TNF-α, IL-17, IL-23) not only drive osteoclast differentiation by upregulating RANKL expression on osteoblasts and stromal cells—accelerating trabecular bone loss—but also stimulate aberrant osteoblast activity via pathways such as Wnt/β-catenin, resulting in syndesmophyte formation and spinal ankylosis [2,11,12].

Beyond its skeletal actions, calcitriol modulates the immune response by shifting T-cell differentiation away from pro-inflammatory Th1 and Th17 phenotypes toward regulatory T-cells and Th2 responses, thereby reducing cytokine production (e.g., IL-17, IL-6, TNF-α) [13]. In addition, calcitriol inhibits NF-κB activation—a key driver of inflammation—further mitigating inflammatory processes [14]. Many immune cells, including dendritic cells and T-cells, can locally convert 25(OH)D to calcitriol, which acts in an autocrine and paracrine manner to fine-tune the inflammatory response at sites such as the entheses [15].

Maintaining serum 25(OH)D levels within the optimal range (typically 30–50 ng/mL) is essential for calcium homeostasis and immune modulation [16]. Adequate calcitriol levels promote efficient intestinal calcium absorption, support balanced bone remodeling via VDR activation [6], and help maintain immune homeostasis by modulating T-cell responses. In patients with axSpA, chronic inflammation may reduce vitamin D bioavailability, necessitating higher supplementation doses or more frequent monitoring [14].

Given that vitamin D enhances calcium absorption, its supplementation is often combined with adequate calcium intake—a strategy that is particularly important in osteoporotic patients. Moreover, vitamin K2 may work synergistically with vitamin D to direct calcium deposition into the bone matrix rather than soft tissue, thereby potentially improving both bone density and quality [17].

Practical Recommendations and Individualized Therapy

- Baseline assessment: Evaluate serum 25(OH)D, calcium, and PTH levels before initiating supplementation to tailor the dose to the patient’s needs.
- Monitoring and adjustments: Regular monitoring of vitamin D levels, bone turnover markers, and inflammatory indices (e.g., CRP, ESR) allows for dosage adjustments to optimize outcomes [18].
- Adjunctive lifestyle modifications: Weight-bearing exercise, adequate sunlight exposure, and a diet rich in calcium and vitamin D are essential to support bone health [18].
- Vitamin D is integrally involved in both skeletal and immune health. Its active metabolite, calcitriol, is vital for optimal calcium absorption, bone matrix integrity, and the modulation of inflammatory processes. In conditions such as osteopenia, osteoporosis, and axial spondyloarthritis, vitamin D deficiency can trigger secondary hyperparathyroidism and impaired bone remodeling, thereby increasing fracture risk and contributing to abnormal bone formation. Future research should focus on the following:
- Mechanistic studies: Detailed explorations of VDR activation, NF-κB inhibition, and the modulation of the RANK/RANKL/OPG axis in both normal and inflamed bone tissue.
- Randomized controlled trials: Well-designed studies to determine optimal dosing; the long-term benefits of combination therapy with vitamin D, calcium, and vitamin K2; and impacts on clinical outcomes such as fracture rates and disease activity in axSpA.
- Personalized supplementation strategies: Developing individualized treatment protocols based on genetic predispositions, baseline vitamin D status, and inflammatory profiles to maximize benefits and minimize risks [19].

Radiofrequency Echographic Multi-Spectrometry (REMS) is an innovative, non-ionizing ultrasound-based technique designed to assess bone mineral density (BMD) and bone quality. Unlike conventional B-mode ultrasound imaging, REMS captures raw radiofrequency signals that contain rich spectral information. This information is processed by comparing the acquired spectra with pre-established reference models corresponding to healthy or osteoporotic bone. In doing so, REMS provides not only BMD values (along with T- and Z-scores) but also additional indices—such as a fragility score—that may reflect the underlying microarchitectural integrity of bone tissue. Although this approach holds promise, its performance can be influenced by factors like probe positioning and signal quality, making standardization and operator training critical for reliable outcomes [20].

In clinical practice, REMS offers several potential advantages over traditional dual-energy X-ray absorptiometry (DXA). Its radiation-free nature and portability make it especially attractive for use in populations where exposure to ionizing radiation is a concern, such as in children, pregnant women, and patients requiring frequent monitoring. Studies have demonstrated a good diagnostic concordance between REMS and DXA for the detection of osteoporosis, and REMS has shown potential in assessing fracture risk by evaluating bone quality parameters that DXA might miss [21,22]. However, real-world applications have revealed challenges—including variable scan quality and operator dependency—that may affect its reproducibility and overall diagnostic accuracy [20,23].

Despite these promising findings, critical gaps remain in our understanding of REMS. Many studies involve limited populations, and issues such as inter-operator variability, machine calibration, and the influence of patient-specific factors (e.g., obesity or skeletal deformities) on signal quality have yet to be fully addressed. Furthermore, while REMS can provide additional information on bone quality via indices like the fragility score, further validation is needed to determine how these measures correlate with long-term fracture risk. The technology’s sensitivity to artifacts and its reliance on accurate region-of-interest selection underscore the need for standardized protocols and rigorous quality control measures [23].

Looking ahead, future research should focus on large-scale, multicenter studies that evaluate REMS across diverse demographic groups. Such studies are essential to establish robust normative databases, refine the algorithms used for spectral analysis, and integrate advanced data analytics (such as machine learning) to improve the precision of bone quality assessment. Moreover, cost-effectiveness analyses and long-term outcome studies will be crucial in determining whether REMS can reliably complement—or even replace—DXA in routine clinical practice [24].

In addition to its applications in primary osteoporosis, REMS holds the potential for monitoring bone health in patients with spondyloarthritis (SpA) who are undergoing vitamin D treatment. SpA is often accompanied by chronic inflammation that can adversely affect bone density and quality. Vitamin D supplementation is widely used in SpA to support bone mineralization and modulate inflammatory processes. With its ability to provide real-time, radiation-free evaluations of bone status, REMS can be employed to perform serial assessments of BMD and bone quality in these patients. This enables clinicians to track the efficacy of vitamin D therapy over time and to adjust treatment plans accordingly, potentially reducing the risk of fragility fractures and improving overall skeletal health in the SpA population [22,24].

In summary, REMS represents a promising advancement in non-invasive bone assessment by providing both quantitative and qualitative information without exposing patients to radiation. While initial clinical validations are encouraging, further research is needed to address the issues of standardization, operator training, and long-term outcomes. Large-scale, longitudinal clinical trials will be key to establishing REMS as a robust, widely accepted tool in the management of osteoporosis, spondyloarthritis, and other metabolic bone diseases [17,20,24].

The main objective of the present study is to provide an insight in the usefulness of the fragility score (FS) obtained through REMS analysis, as a marker of bone quality, by comparing its variability under vitamin D supplementation in AxSpA patients. The FS is an evaluation that has not been thoroughly studied in regards to bone health and the study could represent a starting point for further research. Furthermore, we tried to compare the utility of vitamin D supplementation in regards to muscle strength and fall prevention, areas that are still under debate in different scientific endeavors.

## 2. Materials and Methods

Many clinical studies have followed up on BMD variation under vitamin D supplementation by using DXA as a gold-standard form of evaluation. The scientific literature is lacking in this aspect, particularly regarding the usefulness of REMS in BMD follow-up and the importance of the fragility score (FS) in the previously mentioned conditions. The main objective of this to study was to determine whether BMD follow-up and FS follow-up are relevant in a group of patients with axial spondyloarthritis and whether REMS could have clinical applicability.

Ethics committee approval was not required for this study, as national legislation states that only interventional studies with medical products or medical devices that could harm the integrity of individuals require such approval.

An observational, prospective, minimally interventional study was performed. A cohort of 76 patients with confirmed AxSpA was recruited in order to meet the above objective. These individuals were recruited from two healthcare centers. All patients were recruited during their regular check-ups, conditioned by the following inclusion criteria:

- Having a positive AxSpA diagnosis based on the ASAS classification criteria (2009) [25];
- Having completed standard bloodwork specific for AxSpA (must include inflammatory markers like ESR and CRP);
- Having completed lumbar spine X-rays (at least lateral incidence);
- Having completed blood tests detecting the presence of HLA-B27 at any time in the past;
- Not diagnosed with any other pathology that could influence bone metabolism (e.g., diabetes mellitus, hyperparathyroidism, hypothyroidism);
- Not taking medication that reduces bone density (glucocorticoid therapy orally, intravenously, or intramuscularly);
- Having read, understood, and signed the informed consent form;
- Having a recent test result for 25-OH-Vitamin D, or performing the test within a month of the first visit;
- Agreeing to biannual follow-up visits for 18 months.

A total of 100 patients were initially added to the database (between July 2021 and January 2022). Of these, 13 had diabetes mellitus diagnosis, 5 had recently received glucocorticoid therapy (within 6 months of evaluation), and 6 had never undergone HLA-B27 testing. The remaining 76 patients continued in this study (starting March 2022), and the database was updated. All patients were recently diagnosed and did not have criteria for initiating any classical synthetic DMARDs treatment. After the patients’ inclusion, their anamneses provided information about their muscle strength (using a visual analog scale of 0–100, where 0 indicates low muscle strength and 100 indicates no issues with muscle strength), their number of falls originating from muscle weakness, and the consequences of these falls. A REMS analysis was performed using the Echolight© machine that was present in the two institutions. The evaluation was conducted for standard sites such as the lumbar spine (L1 to L4) and both hips. Spinal analysis provides automatic measurement of the FS, a marker for bone fragility, used for follow-up in these individuals. Afterward, a single-blind study randomization was performed to split the cohort into two study groups: one with vitamin D supplementation and one without. It is important to mention that patients with deficits were automatically included in the supplementation branch. Vitamin D supplementation was offered, and the study team covered the costs. The option for vitamin D was cholecalciferol, in gelatinous capsules, with a dosage of 2000 UI/day. The patients were instructed to take a capsule in the morning after 15 min of fasting after breakfast. Based on national ethics laws, there was no need for approval from an ethics committee since vitamin D is not an experimental medication. Moreover, REMS is a non-irradiative diagnostic procedure that has been proven in multiple clinical studies and is not harmful in any way.

The first follow-up visits occurred after 6 months (visit 1) when the patients returned for regular check-ups. The REMS examination was repeated during these visits. No patient had any issues regarding vitamin D supplementation. The VAS for muscle strength was repeated, and an updated anamnesis was obtained for falls.

At the second follow-up (12 months from visit 0), REMS was repeated, and standard blood tests, including ESR and CRP, were performed during regular clinical check-ups. The VAS and anamnesis were repeated once again.

The last visit (visit 3) repeated all examinations from visit 0 (REMS, anamnesis, VAS for muscle strength and vitamin D evaluation, standard blood test).

After the database was constructed, descriptive statistics were obtained using Excel and Minitab statistical software, and a one-way ANOVA was used to analyze the data obtained during the aforementioned follow-ups.

It is important to mention that REMS evaluations automatically include a calculation of the fragility score (FS). For the purposes of the present study, the FS for the spine measurement was compared during follow-ups.

A statistical analysis was performed with MiniTab v.20 (MiniTab LLC) by applying a one-way ANOVA test for variance across the follow-ups. Basic descriptive statistics were obtained using 2-sample *t*-tests.

## 3. Results

**Figure 1:**
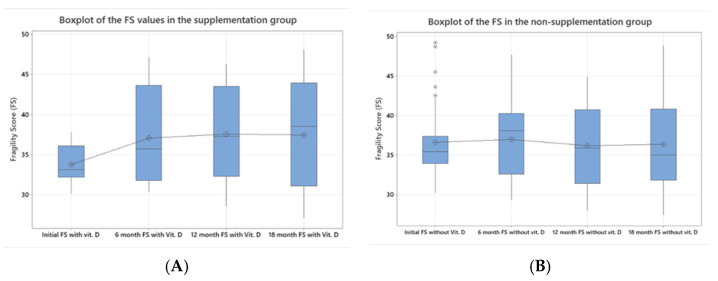
Boxplots comparing the evolution of the FS in the two study groups. (**A**) While initially there was an increase in the FS in the supplementation group, at 6 months, a more stable value was observed. (**B**) In the non-treatment group, there was no significant change over time in regards to the FS group with (*) representing a group of 5 individuals with higher FS scores, beyond the whiskers range of 30.2-42.3 during statistical testing (one-way ANOVA, *p* = 0.008 in the supplementation group and *p* = 0.924 in the other).

After randomization, the two groups were evenly distributed, with very similar mean values for the fragility score (FS). However, a great difference was observed in the maximum values (93.70 for the treatment group versus 49.20 for the non-treatment group). Two more patients were observed to have very high values compared to the maximum of the non-treatment group; these individuals were excluded from the statistical analysis of variance in order to avoid anomalies. Two-sample *t*-tests were performed to assess the study group and treatment groups, with *p*-values of <0.005 (Table 1). These patients were observed separately to check their response to vitamin D treatment. Afterwards, one-way ANOVA testing was performed for both groups to observe the evolution of the FS over time. For the supplementation group, there was a slight initial increase in fragility, but after the 6-month threshold, the FS seemed to come to a standstill (*p* = 0.008, Figure 1A). In the non-treatment group, no significant increase was seen in regard to the FS, with results following a more random distribution (*p* = 0.924, Figure 1B).

**Figure 2:**
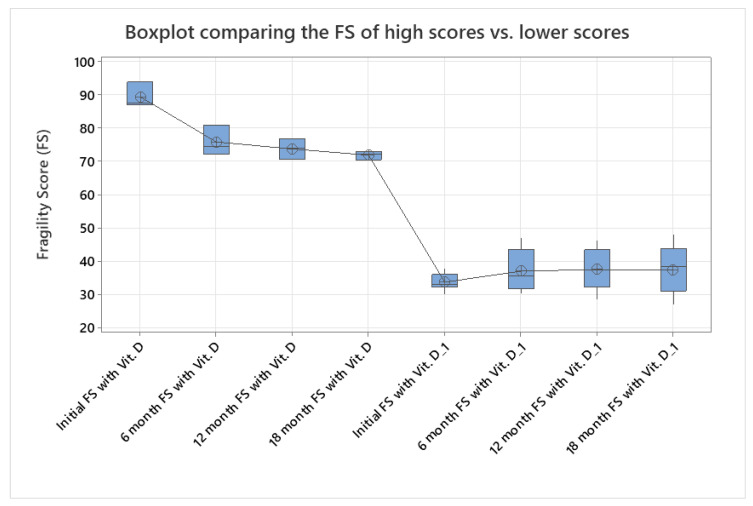
Differences seen between patients with lower initial FS values and higher initial FS values in the course of this study. Patients with higher FS values seemed to benefit more from vitamin D supplementation, with a clear reduction in their FS values (one-way ANOVA, *p* < 0.005).

When the higher FS values (among those who received vitamin D supplementation) were compared with those for the main supplementation group, an interesting result was observed. The higher FS values seemed to indicate a significant reduction in fragility compared to the lower values observed for the standard group (one-way ANOVA, *p* < 0.005, Figure 2).

**Figure 3:**
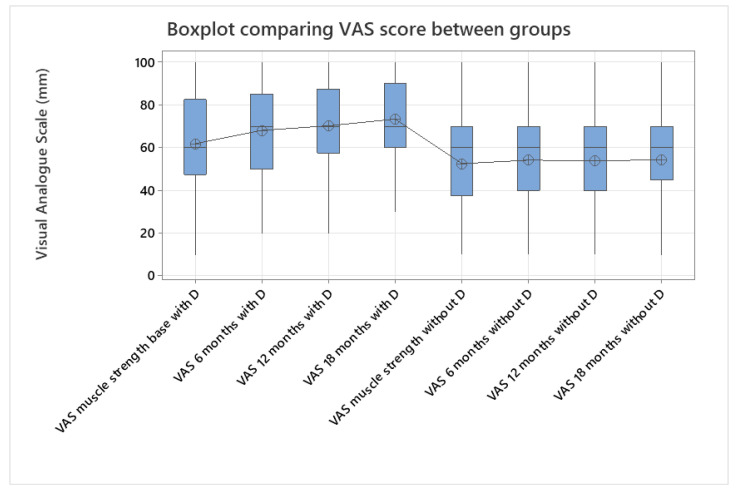
Evolution in time of muscle strength measured using a VAS. A subjective increase in muscle strength can be seen in the supplementation group, which suggests a possible influence of vitamin D on muscle health in AxSpA (one-way ANOVA, *p* < 0.005).

Afterward, the VAS scores were compared by applying the same one-way ANOVA test to assess the study groups, and a slight increase in muscle strength was observed in the supplementation group (*p* < 0.005, Figure 3). Eight patients had mild vitamin D deficits at the start of this study, but at the end of this study, there were no patients with deficits. Three patients reported falls before the randomization, though without any complications, such as fractures or co-motions; during the follow-up period, no new events were recorded in the patients’ anamneses.

During follow-ups, no new treatments were prescribed, as well as no surgical interventions done, as they were not required. The main patient treatment relied on kinetotherapy and NSAIDs for pain control, strictly when needed.

## 4. Discussion

The results add to existing literature regarding the usefulness of vitamin D in patients with AxSpA. The high frequency of vitamin D deficiency discovered by Shevchuk et al. was not observed by the present study, possibly due to the short disease duration for the present examination groups (43.2% compared to only 10.81%, but with a difference of 9 years of disease duration) [26]. Furthermore, the paradox of osteoporosis occurring in patients with a disease characterized by excess bone formation can be explained by the different mechanism affecting bone health, a key point where the REMS FS can shed more light [27]. The increase observed in the FS, especially in patients with higher initial FS values, suggests that proper supplementation with the correct form of vitamin D can reduce overall bone fragility in the lumbar spine in patients with AxSpA by influencing not only elements of bone mineralization, but also regarding bone quality, as mentioned by Żuchowski et al., underlying the need for further study of REMS technology in these radiological AxSpA [28]. These results can be interpreted through cholecalciferol’s impact on bone health but also through its immunomodulatory effects, especially in patients with impaired immune response, but no relevant clinical studies could be found in scientific literature regarding the impact of vitamin D on bone quality in AxSpA. To add to this, vitamin D does not seem to have any impact on pain or disease activity in AxSpA patients [29]. In individuals with a low FS, no significant reduction in the FS was observed during the 1.5-year study period. Longer studies on larger populational groups may be required. In regard to muscle strength, the increase under vitamin D supplementation can be interpreted as either an actual effect of the medication or the placebo effect. Similarly, a systematic review by Lars Rejnmark showed that many studies lacked in proving the actual evidence of how vitamin D could improve muscle health, concentrating more on showing in most studies the increase in strength, function and body sway, while others provided no clear evidence [30]. A thorough study of different populational groups with different sociological and psychological attributes would shed more light on this matter. A decrease in falls was observed, but there were few to begin with and further recruitment of individuals into the study could determine the validity of this information, since there is already some evidence from a recent meta-analysis showing that vitamin D supplementation with doses ranging from 700 to 2000 IU can help in this matter [31].

Unfortunately, the present study has some important limitations that need to be referred to in the future, such as the low number of individuals enrolled, the short period of time for follow-up, and the lack of any csDMARD or biological DMARD treatment. The study team plans on expanding this research to cover the current limitations and provide more clear results in the future, taking into consideration multicenter and multipopulational studies.

## 5. Conclusions

Overall, this study proves that the lumbar FS obtained through REMS evaluation could be a valuable tool in the follow-up of patients with AxSpA. Furthermore, there seems to be evidence that vitamin D supplementation can slightly increase muscle strength and thus reduce the risk of falls in patients with AxSpA.

## Figures and Tables

**Table 1:** Descriptive statistics comparing the main characteristics of the whole group, as well as baseline values for the two study treatment branches (^a^ one-sample *t*-test, *p* < 0.005).

	Male (n = 59)	Female (n = 17)
Age mean (range) ^a^	37.22 (20–65)	36.06 (22–49)
Underweight (%)	0 (0%)	0 (0%)
Normal weight (%)	10 (16.949%)	5 (29.41%)
Overweight (%)	31 (52.54%)	10 (58.82%)
Grade I obesity (%)	18 (30.508%)	2 (11.76%)
Vitamin D mild deficit (n = 8, 10.81%) ^a^	3 (5.08%)	5 (29.41%)
**All (n = 76)**	**Treatment group (n = 38)**	**Non-treatment group (n = 38)**
Initial FS ^a^	38.15 (30.10–93.70)	36.589 (30.20–49.20)
Initial muscle strength VAS ^a^	61.89 (10–100)	52.57 (10–100)
Initial number of falls	2	0

## Data Availability

The data presented in this study are available on request from the corresponding author.
